# Sports and Recreation-Related Wrist Fractures: An Epidemiological Study

**DOI:** 10.7759/cureus.62177

**Published:** 2024-06-11

**Authors:** Gabriel I Onor, Alec Kellish, Michael Chang, Lilah Fones, Tyler Henry, Margaret Pennington, Daniel A Nemirov, Sommer Hammoud, Pedro K Beredjiklian

**Affiliations:** 1 Department of Orthopaedic Surgery, Thomas Jefferson University Hospital, Philadelphia, USA; 2 Division of Hand Surgery, Rothman Orthopaedic Institute, Philadelphia, USA; 3 Division of Sports Medicine, Rothman Orthopaedic Institute, Philadelphia, USA

**Keywords:** sports-related injuries, epidemiology, distal radius, hand surgery, wrist fractures

## Abstract

Background

Wrist fractures have increased over the past several decades. The objective of this study was to identify all-cause and sports-related incidence rates of wrist fractures presenting to emergency departments (EDs) in the United States (U.S.) from 2013 to 2022. A secondary aim of the study was to identify if wrist fractures significantly decreased during 2020.

Methodology

The National Electronic Injury Surveillance System database was queried to identify the number of wrist fractures presenting to U.S. EDs from 2013 to 2022. Incidence rates in 100,000 person-years were calculated by sport, age, sex, and year.

Results

From 2013 to 2022, there were 2,027,131 wrist fractures evaluated at U.S. EDs. Injuries peaked in the 10-14-year-old age group, followed by the 5-9 and 85+-year-old age groups. In total, 1,096,598 were sustained during sports and recreation. Cycling, playgrounds, and skateboarding were the leading sports and recreation-related activities. Sports-related wrist fractures followed a unimodal distribution peaking in the 10-14-year-old age group. Females sustained 52% of wrist fractures overall but only 39% of sports-related wrist fractures. All-terrain vehicle and skateboarding-related wrist fractures significantly increased over the study period. Playground and soccer-related wrist fractures significantly decreased in 2020.

Conclusions

All-cause wrist fractures presenting to U.S. EDs significantly increased from 2013 to 2022 though sports-related wrist fractures did not. Pediatric males and elderly females are most at risk for wrist fractures overall while sports-related wrist fractures predominate in the pediatric population. Youth sports and recreation officials should be aware of the risks to mitigate the incidence of sports-related wrist fractures.

## Introduction

Fractures about the wrist pose a significant detriment to quality of life and may limit the ability to participate in activities of daily life as well as sports and recreation. Wrist fractures are a broad category and include fractures of the carpal bones, the distal radius, and/or the distal ulna. The scaphoid is the most commonly fractured of the carpal bones, accounting for over 50% of carpal fractures [1].

Several studies have reported an increasing incidence of distal radius fractures over the past few decades [2,3]. Moreover, recent literature has demonstrated increasing rates of upper extremity and, specifically, wrist fractures presenting to United States (U.S.) emergency departments (EDs) [4-6]. However, there is a dearth of information regarding the epidemiology of sports and recreation-related wrist fractures as well as the impact of the COVID-19 pandemic on the incidence of wrist fractures.

The purpose of this study was to identify the overall incidence of wrist fractures presenting to U.S. EDs from 2013 to 2022 as well as the incidence of those sustained as a result of sports and recreation. This study additionally aimed to investigate the relationship of sex and age on the incidence of wrist fractures. We hypothesized that wrist fractures would follow a bimodal distribution overall and in sports with the highest incidence in the pediatric and elderly populations. We also hypothesized that wrist fractures would decrease significantly during 2020 in light of the COVID-19 pandemic.

## Materials and methods

The National Electronic Injury Surveillance System (NEISS) database was queried to identify wrist fractures based on body part code 34 (wrist) and diagnosis code 57 (fracture) during the 10-year period from 2013 to 2022. The NEISS is a publicly available database published by the U.S. Consumer Product Safety Commission. The database relies upon injury data from 100 representative samples of U.S. EDs to provide national estimates of injury rates. The NEISS sample is stratified and takes into account geographic location and ED size. The representative, weighted sample data for any given diagnosis are then extrapolated to provide nationwide estimates to approximate the number of presentations to the 5,000+ U.S. hospitals.

Body part code 34 and diagnosis code 57 were input into the NEISS database to provide wrist fracture estimates presenting to U.S. EDs from 2013 to 2022. The query was then repeated with the same body part and diagnosis codes to obtain estimates for the demographic variables of concern. Wrist fractures were then analyzed by demographic and epidemiological data, including age, sex, and yearly data of injury estimates. Incidence rates (IRs) were calculated in 100,000 person-years. U.S. Census data was used for yearly population estimates to calculate IRs. Moreover, 95% confidence intervals (CIs) of IRs were calculated based on CIs of injury estimates provided by NEISS.

All statistical analysis was performed in Microsoft Excel (Microsoft Corp., Redmond, WA, USA). Chi-square tests were performed to identify differences by sex and age. Regression analyses were performed to identify trends in yearly injury estimates. Grubbs statistics were calculated to identify significant outliers in wrist fracture incidence with particular concern for the year 2020. Originally described by Grubbs in 1969, the formula to calculate the Grubbs statistic is the absolute value of the data point in question - the average of the data set divided by the standard deviation of the data set [7]. The Grubbs statistic associated with a p-value of 0.05 or less for a data set of 10 individual counts is 2.18. Thus, the Grubbs statistics that were greater than or equal to 2.18 were deemed to represent statistically significant outliers for the year 2020. Statistical significance was set at a p-value less than 0.05.

## Results

From 2013 to 2022, an estimated 2,027,131 wrist fractures presented to U.S. EDs. This corresponds to an IR of 61.4 wrist fractures per 100,000 person-years (95% CI = 49.8-73.1). Among these wrist fractures, 1,096,598 wrist fractures were sustained as a result of sports and recreation (IR = 33.2; 95% CI = 26.4-40.1).

Overall yearly trend

**Figure 1:**
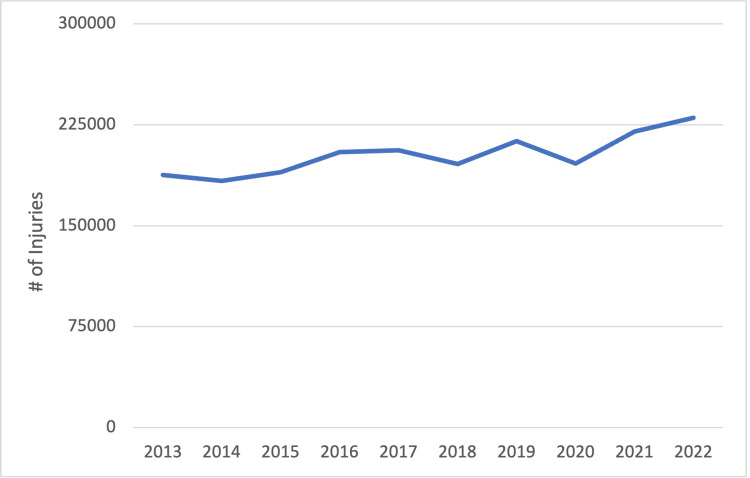
All-cause wrist fractures.

**Figure 2:**
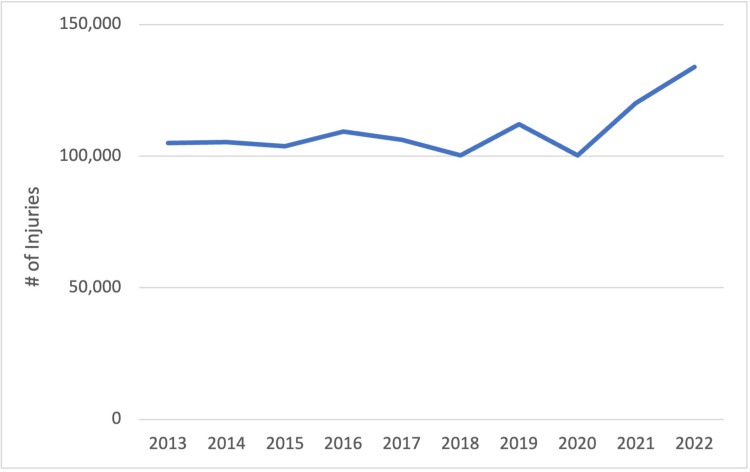
Sports-related wrist fractures.

There was a significant increase in all-cause wrist fractures during the study period (p < 0.05) (Figure 1). The year 2020 was not a significant outlier with a Grubbs statistic of 0.43. There was no significant increase in sports-related wrist fractures during the study period (Figure 2). Across all sports, 2020 also was not a significant outlier with a Grubbs statistic of 0.90.

Incidence by sports and recreation

Cycling (IR = 4.8; 95% CI = 3.7-6.0), playgrounds (IR = 4.7, 95% CI = 3.5-5.8), and skateboarding (IR = 3.7, 95% CI = 2.8-4.6) contributed to the most sports and recreation-related wrist fracture presentations, respectively. The top 10 sports and recreation activities are listed in Table 1.

**Table 1:** Top 10 sports-related wrist fracture incidence overall. ATVs = all-terrain vehicles; IR = incidence rate

Sports/Recreational Activity	IR
Bicycles and accessories	4.85
Playground equipment	4.68
Skateboards, scooters, hoverboards	3.70
Skating, all kinds	2.80
Soccer	2.35
Football	2.23
Basketball	2.17
ATVs, mopeds, minibikes, etc.	1.84
Miscellaneous sports	1.44
Exercise and equipment	1.44

Yearly sports trend

The top 10 sports for wrist fractures that had statistically significant increases during the study period were all-terrain vehicles (ATVs) (p < 0.05) and skateboarding (p < 0.05). Playgrounds and soccer had statistically significant outliers of injuries in 2020 with Grubbs statistics of 2.58 and 2.44, respectively. The remaining sports/activities did not have significant overall trends or significant outliers in 2020.

Age distribution

**Figure 3:**
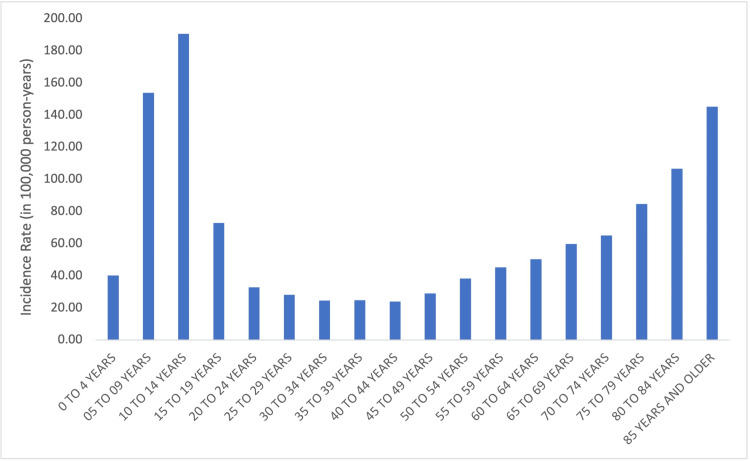
Overall incidence rates of wrist fractures by age.

The incidence of overall wrist fractures was highest in the 10-14-year-old age group (IR = 190.5, 95% CI = 149.6-231.4) followed by the 5-9-year-old (IR = 153.9, 95% CI = 118.3-189.5) and 85+-year-old (IR = 145.3, 95% CI = 110.2-180.4) age groups. The incidence of overall wrist fractures varied significantly by age group (p < 0.05) (Figure 3).

**Figure 4:**
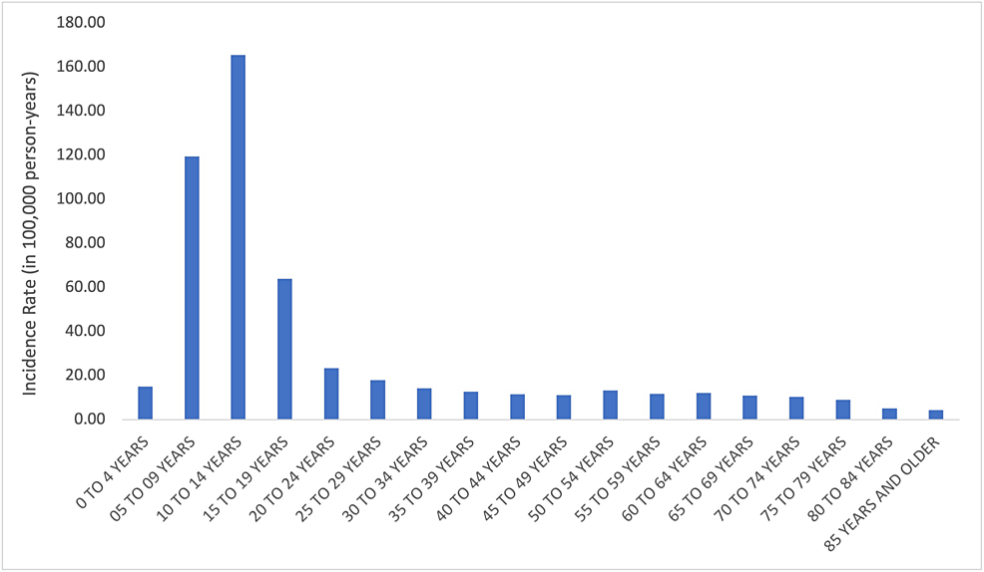
Sports-related wrist fractures by age.

In sports and recreation-related wrist fractures, the incidence was the highest in the 10-14-year-old age group (IR = 165.7, 95% CI = 129.6-201.9), followed by the 5-9-year-old (IR = 119.6, 95% CI = 91.4-147.8) and 15-19-year-old (IR = 64.0, 95% CI = 51.5-76.5) age groups. The incidence of sports-related wrist fractures varied significantly by age group (p < 0.05) (Figure 4).

The number of wrist fractures sustained in 5-9-year-olds during 2020 was the only age group with a significant outlier that year with a Grubbs statistic of 2.37. The age groups with statistically significant increases across the study period were 0-4, 25-29, and all groups 55 years and older (p < 0.05).

Wrist fractures by sex

The incidence of wrist fractures overall was 63.2 per 100,000 person-years (95% CI = 50.9-75.5) in females and 59.2 (95% CI = 48.3-71.0) in males. The incidence of wrist fractures overall varied significantly by sex (p < 0.05).

**Figure 5:**
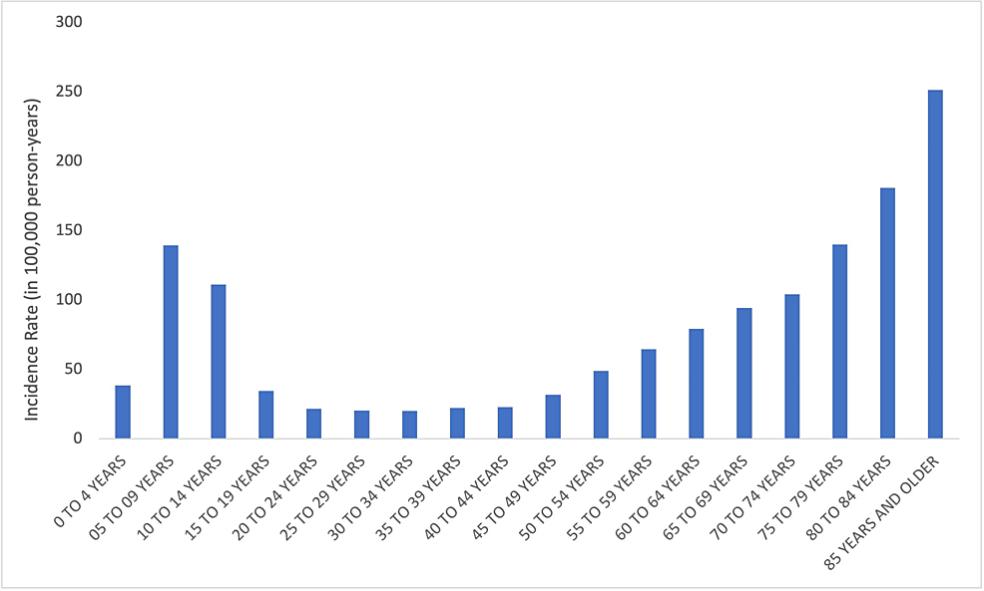
All-cause incidence of wrist fractures in females.

**Figure 6:**
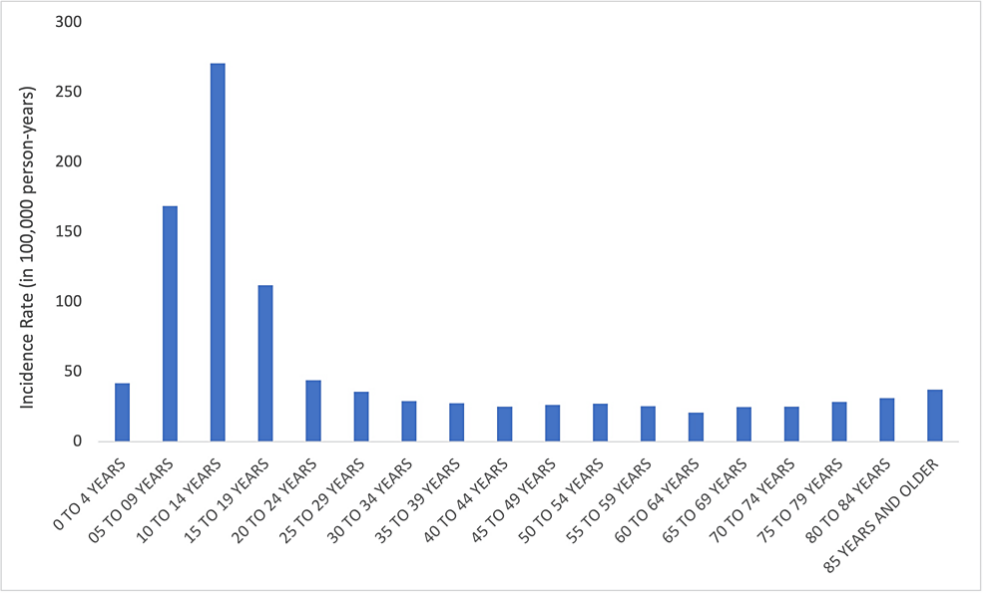
All-cause incidence of wrist fractures in males.

The incidence of sports-related wrist fractures was 25.5 (95% CI = 20.0-31.0) in females and 41.1 (95% CI = 32.7-49.5) in males. The incidence of sports-related wrist fractures differed significantly by sex (p < 0.05). The top 10 leading sports by sex may be found in Table 2 and Table 3. The incidence of wrist fractures in females peaked in the 85+-year-old age group and the 10-14-year-old age group in males (p < 0.05) (Figures 5, 6).

**Table 2:** Top 10 sports-related wrist fracture incidence in males. ATVs = all-terrain vehicles; IR = incidence rate

Sports	IR
Bicycles and accessories	6.58
Skateboards, scooters, hoverboards	5.24
Playground equipment	5.24
Football	4.32
Basketball	3.73
Soccer	3.35
ATVs, mopeds, minibikes, etc.	2.82
Skating, all kinds	1.62
Snow skiing	1.38
Miscellaneous sports	1.03

**Table 3:** Top 10 sports-related wrist fracture incidence in females. ATVs = all-terrain vehicles; IR = incidence rate

Sports	IR
Playground equipment	4.14
Skating, all kinds	3.96
Bicycles and accessories	3.15
Skateboards, scooters, hoverboards	2.18
Exercise and equipment	1.89
Miscellaneous sports	1.83
Soccer	1.37
Snow skiing	0.90
ATVs, mopeds, minibikes, etc.	0.88
Trampolines	0.75

## Discussion

Fractures about the wrist have increased steadily during the past several decades [2,3,5,6]. Our study was in alignment with the previous literature and found that all-cause wrist fractures increased significantly from 2013 to 2022. However, sports-related wrist fractures in isolation did not increase significantly during the same period. The authors would have expected that sports-related wrist fractures would also increase during the study period, especially with the rise in popularity of recreational activities such as pickleball among older age groups.

Overall, this study found a bimodal distribution of wrist fractures with one peak during adolescence and a peak in elderly wrist fractures occurring in the 85+-year-old group. This finding is in agreement with a 2023 study which also utilized the NEISS database and found an incidence of upper extremity injuries overall peaking in the 85+-year-old age group [4], but contrasts an earlier report from 2000 by Da Laet and Pol that the incidence of elderly wrist fractures peaked in the 60-70-year-old age group [8]. Together, this suggests that though there remains an increase in wrist fractures in the elderly relative to younger adults, there is a shift in the peak to those greater than 85 years old.

The mechanism of wrist fractures differs with age. Younger patients are more likely to experience higher energy, sports, and recreation injuries while older patients are more likely to experience standing height fall-related wrist fractures [9-13]. When only sports and recreation-related wrist fractures are analyzed, there is a unimodal distribution with a peak in the 10-14-year-old age group without a second peak in the elderly (Figures 3, 4). To our knowledge, this is the first study to identify sports-related wrist fracture incidence across all age groups with the NEISS database.

Moreover, in the elderly population, falls have been demonstrated to be the leading cause of distal radius fractures [6,8]. As the elderly population grows in the U.S., the incidence of wrist fractures may continue to grow accordingly. Our study demonstrated a statistically significant increase in wrist fractures in all age groups above 55 years during the study period. Fall prevention initiatives will be critical in efforts to slow this increasing rate.

Trends in the incidence of wrist fractures also vary by sex. Wrist fractures in females followed a bimodal distribution with one smaller peak in adolescence and one larger peak in the 85+-year-old age group (Figure 5). In contrast, wrist fractures in males followed a unimodal distribution with a peak in the 10-14-year-old age group (Figure 6). This indicates that in the pediatric population, males are most at risk for wrist fractures, while in the elderly population, females are more at risk. These findings are in agreement with previous literature which points to higher rates of wrist fracture in pediatric males than females and higher rates in elderly females than elderly males [14,15].

The COVID-19 pandemic peaked in 2020 which was the eighth year of our 10-year study period. We expected that all wrist fractures overall and across all sports would decline significantly during the year 2020. However, we found that only playground-related and soccer-related wrist injuries declined significantly during that year. For playgrounds, it follows that these injuries would decline significantly during this year due to the widespread closure of parks and recreation centers. This could also explain the decrease in soccer-related wrist fractures during 2020.

ATV and skateboarding-related wrist fractures increased significantly over the study period. Both have been implicated in previous studies as having led to increased ED presentations as a result of head injuries and ankle injuries, respectively, both during and after the COVID-19 pandemic’s height [16,17]. This may be a reflection of the fact that these activities are less regulated and therefore were not prohibited to the same degree during the height of the COVID-19 pandemic. Additionally, these activities may be performed on an individual basis in compliance with social distancing recommendations. Thus, more people may have participated in these activities leading to an increase in the number of wrist fractures as well. Furthermore, as these activities grow in popularity post-COVID-19, it may follow that ATV and skateboarding-associated wrist fractures may continue to increase.

This study is not without limitations. First, this study relies upon accurate and appropriate coding of injuries by NEISS. Fracture-dislocations may in some instances be coded as dislocations alone which would lead to underreporting of these more severe fractures. Another inherent limitation of the NEISS database is that national estimates of injuries are provided using actual ED data from 100 sample U.S. EDs. It is possible that the actual nationwide population data may not closely follow NEISS national estimates. Additionally, NEISS coding does not delineate between osseous structures in the wrist. Therefore, body part code 34 (wrist) may include injuries spanning the distal radius, the distal ulna, and the entirety of the carpus. This somewhat limits the applicability of the study as the included injuries might range from non-operatively managed triquetral avulsion fractures to displaced intra-articular fractures of the distal radius. Finally, the NEISS database does not account for a likely small percentage of patients who sought care for their injury outside of an ED.

## Conclusions

All-cause wrist fractures increased significantly from 2013 to 2022, though there was no significant increase in sports-related wrist fractures during this period. COVID-19 did not lead to a significant decrease in wrist fractures during 2020 overall, though there were significant decreases specifically in playground and soccer-related wrist fractures in 2020. Wrist fractures overall followed a bimodal distribution while there was a unimodal distribution for sports-related wrist fractures peaking in the pediatric population. Moreover, in the pediatric population, males sustained greater rates of wrist fractures, while in the elderly population, females sustained more wrist fractures. In the elderly population, public health measures should be undertaken to slow the trend toward an increase in fall-related distal radius fractures. Youth sports and recreation officials should be aware of the risk of pediatric wrist fractures and counsel young athletes accordingly with preventative measures.
